# Perceptions and experiences of community pharmacists about weight loss dietary supplements: a qualitative study

**DOI:** 10.1186/s40780-025-00497-4

**Published:** 2025-10-22

**Authors:** Leena Ali Abdul-Ameer, Ehab Mudher Mikhael, Pei Lin Lua

**Affiliations:** 1 Diabetic and endocrine center – Al-Zahraa Teaching Hospital, Wassit, Iraq; 2 Clinical Pharmacy Department, College of Pharmacy, University of Baghdad, Baghdad, Iraq; 3 Faculty of Pharmacy- Universiti Sultan Zainal Abidin (UniSZA), Terengganu, Malaysia

**Keywords:** Weight loss, Dietary supplements, Pharmacists' perception, Pharmacists' experience, Iraq

## Abstract

**Background:**

Obesity is a global, complex, multifactorial disease that poses a direct threat to human health. Many dietary-supplements (DS) are marketed for weight loss. Despite the high demand for DS by obese individuals, the effectiveness and safety of most products are subjected to substantial controversy.

**Aim:**

To explore the perceptions and experiences of pharmacists about weight loss DS (WLDS).

**Methods:**

A qualitative study was conducted in Wassit, Iraq, using face-to-face, individual interviews with pharmacists with ≥ 6 months of working-experience in community pharmacies. Interviews were conducted till reaching the saturation point. Data were analyzed by thematic-analysis approach.

**Results:**

Interviews were conducted with 25 pharmacists. Most participating pharmacists considered WLDS to be ineffective with questionable safety and quality. Most participants believed that the rate of prescribing WLDS is high especially for patients suffering from infertility and chronic diseases. Two-thirds of study participants were neglecting patient-education about the dispensed WLDS. Patient-education when done is mainly limited to discussing the product side effects. The majority of participating pharmacists did not receive education or training about DS. To enhance pharmaceutical care for customers using WLDS, pharmacists emphasized the need for rigorous assessments of product efficacy, safety, and quality before marketing, along with improved patient education efforts.

**Conclusion:**

Participating pharmacists reported that WLDS products are most commonly prescribed for patients with infertility and chronic diseases due to non-medical factors. Pharmacists express concerns about WLDS, highlighting the need for rigorous assessments before marketing. Gaps in pharmacists’ patient education roles necessitate targeted training focused on enhancing communication skills, evidence-based counseling, and awareness of WLDS risks to improve patient outcomes.

## Introduction

Obesity is a global, complex, multifactorial, and generally preventable disease [1]. Obesity is characterized by abnormal or excessive fat accumulation that directly threatens human health [2]. Obesity is a common problem worldwide, and its prevalence has tripled in the last five decades [1]. In Iraq, obesity prevalence has increased dramatically in the last two decades from 25% to about 43% during the period from 2000 to 2020 [3]. Among the main reasons for this rise is the tendency for a sedentary lifestyle [4] and the increasing consumption of fast food [5, 6].

Unfortunately, obesity is associated with an increased risk of cardiometabolic, respiratory, gastrointestinal, and musculoskeletal diseases, besides malignancy [7]. Lifestyle modification (i.e., calorie restriction and exercise) is essential in managing obesity [8, 9]. However, it is difficult for most individuals to adhere to lifestyle modifications without motivational education programs that can ensure behavioral change and reinforcement of the change [10]. Nonetheless, several medications have now been approved for obesity management, including orlistat, phentermine/topiramate, naltrexone/bupropion, liraglutide, setmelanotide, tirzepatide, and semaglutide [11]. However, most of these medications are expensive and have undesirable side effects [12, 13]; besides, they are not easily accessible to patients without a prescription [13].

Many dietary supplements (DS) are marketed for weight loss [14]. In Iraq, the registration and inclusion of DS in the national list is a relatively straightforward process through the National Committee for the Selection of Herbal Medicines and Food Supplements [15]. Additionally, these supplements can be dispensed in pharmacies without a prescription, a practice that is common in Iraq and many other countries [16]. The easy accessibility and extensive availability of a wide variety of supplements in pharmacies may prompt obese individuals to pursue weight loss treatment through DS [17].

Therefore, the use of herbal dietary supplements like green tea and ginger is very common among Iraqi obese individuals [18]. Besides dispensing OTC products, Iraqi pharmacists can play an important role in the management of obesity and other chronic diseases by referring cases with alarming features to the physician [19], helping physicians in choosing appropriate treatment, and educating patients about their prescribed treatment [20]. Furthermore, many physicians in Iraq, particularly in private clinics rather than public hospitals prefer to prescribe DS for the management of obesity and various other chronic disorders [21–23].

Despite the high demand for DS by obese individuals, their effectiveness and safety are controversial due to the lack of strong clinical evidence [17, 24]. Moreover, many adverse events could occur while consuming these supplements [25], and most were not subjected to quality assessment before being marketed in pharmacies [26]. Unfortunately, information about the DS that are used for weight loss in Iraq is limited.

Given the widespread availability of dietary supplements marketed as products designed to aid in weight reduction (WLDS) in community pharmacies, and the vital role of pharmacists in obesity management through providing appropriate advice and ensuring safe, effective treatment, understanding their perceptions, experiences, and dispensing practices is essential. Therefore, this study aims to gain an in-depth understanding of community pharmacists’ perceptions, experiences, and practices regarding WLDS. Exploring these aspects will help identify areas where pharmacists may require additional support or education, ultimately contributing to the development of targeted interventions and improved regulatory measures to promote safe and effective consumer care.

## Methods

### Study design and setting

Given the complexity and context-specific nature of the topics—namely, pharmacists’ perceptions, experiences, and dispensing practices related to WLDS—qualitative methods are well-suited to gather in-depth insights directly from participants, capturing their perspectives in their own words. This approach facilitates a detailed exploration of how pharmacists perceive and manage WLDS in their practice, providing rich, descriptive data that cannot be obtained through quantitative methods, thereby effectively supporting the current research objectives.

A phenomenological qualitative study was performed through face-to-face individual-based interviews with community pharmacists in Wassit, Iraq.

Wassit is a governorate located in eastern Iraq, southeast of Baghdad, and shares a border with Iran. As of 2021, its population was approximately 1,489,631 [27]. Health services across Iraq remain unevenly distributed and are challenged by a significant shortage of health workers, infrastructure, and medical supplies. In this context, Wassit currently lacks any specialized obesity or metabolic treatment centers [28]. According to data from the Syndicate of Iraqi Pharmacists, there are over 550 pharmacies in Wassit, with the majority located in its larger cities.

The study was ethically approved by the ethical committee at the College of Pharmacy, Baghdad University (Ethical Approval Number: RECAUBCP812023Q at 8th Jan, 2023).

The research was conducted in accordance with the Declaration of Helsinki. All participants were informed about the study’s purpose, procedures, and their rights, and only those who provided their verbal informed consent were included in the study.

### Development and validation of the interview guide

The interview guide (Appendix 1) was prepared by study authors based on the ideas in relevant literature [21, 29].

For content validation, the interview guide was sent to a panel of three academic pharmacists with PhD in clinical pharmacy and were experienced in conducting qualitative studies. The experts were asked to rate each question on a 3-point scale: essential, useful but not essential, and not useful. Moreover, the experts were asked to write their comments on the language and wording issues. Lawshe’s approach was chosen to determine the content validity of the interview guide [30]. Fortunately, all experts came to a mutual agreement about the essentiality and relevance of all questions in the interview guide. Meanwhile, a comment about wording issues was received from one expert, and the guide was corrected accordingly.

### Setting and participant recruitment method

A convenient-strategy was used to enroll the participants in this study. According to data from the Syndicate of Iraqi Pharmacists, there were approximately 550 registered pharmacies in Wassit at the time of this study, representing 5% of the total registered pharmacies in Iraq. Because all pharmacists in Iraq are qualified to work in community pharmacies [31], only pharmacists with at least 6 months of working experience in community pharmacies were eligible to participate in this study. Eligible participants were contacted in their pharmacies and informed about the study.

Pharmacists who do not have WLDS in their pharmacies were excluded from the study. For the purposes of this research, WLDS are defined as products marketed to promote weight reduction, available in any dosage form, and typically containing ingredients such as plant extracts, vitamins, or other compounds claimed to aid in weight loss, which are commonly accessible through pharmacies.

Only those who provided their verbal informed-consent were enrolled in this study. Those who accepted to participate in the study and were busy were asked to specify a date for an interview according to their work schedule. To get views from pharmacists working at different regions of Wassit governorate, only one pharmacist from each pharmacy, preferably the pharmacy owner, was allowed to participate in this study. All participants were interviewed in their pharmacies.

### Data collection and analysis

The interviews were guided by semi-structured open-ended questions. Probes were used to elicit further comments when necessary. All interviews were conducted in Arabic and then translated to English by the first author (a high diploma candidate in clinical pharmacy), who received training in conducting qualitative interviews from the second author (PhD in clinical pharmacy with experience in qualitative studies). All interviews were audio-recorded using a mobile recorder. Each interview took approximately 10–20 min to be completed.

The interviews were conducted until the point of saturation was reached (whereby no more new information was acquired). To reach this point the interviews continued from May to July 2023.

All interviews were coded manually by the second author and used for the qualitative data sorting. Codes were grouped to generate themes. The coding procedure was established by a thorough reading of each interview transcript. A codebook was developed to ensure consistent coding across interviews. The obtained data was analyzed using Braun and Clarke’s thematic analysis approach, which relies on the participants’ quotes, codes, and themes [32].

## Results

In-depth interviews were conducted with 25 pharmacists. Most participants were young (*n* = 20;80%) female (*n* = 19;76%) pharmacists. Further details are given in Tables 1 and 2. Study themes are shown in Table 3.

**Table 1:** Demographic data of study participants

Parameter		Value (percent)
Age	25–35 years	20 (80%)
	36–45 years	4 (16%)
	More than 45 years	1 (4%)
Gender	Male	6 (24%)
	Females	19 (76%)
Academic degree	BSc	18(72%)
	High diploma	3(12%)
	MSc	3(12%)
	PhD	1(4%)
Working experience*	Less than 1 year	1(4%)
	1–5 years	11 (44%)
	6–10 years	10 (40%)
	More than 10 years	3 (12%)

* Working experience refers to the total years of active practice in a community pharmacy

**Table 2:** Detailed demographics of study participants

Participant no.	Age (in years)	Gender	Academic degree	Graduated from	Working experience
P1	36	Male	BSc	International University	10 years
P2	28	Female	BSc	National Public University	6 years
P3	26	Female	BSc	National Public University	2.5 years
P4	26	Female	BSc	National Private University	2.5 years
P5	28	Male	BSc	National Private University	6 years
P6	29	Female	BSc	National Public University	6 years
P7	30	Female	BSc	National Public University	7 years
P8	25	Female	BSc	National Public University	1 years
P9	25	Female	BSc	National Public University	1.5 years
P 10	28	Female	High diploma	National Public University	6 years
P 11	28	Female	MSc	National Public University	14 months
P 12	25	Male	BSc	National Public University	1.5 years
P13	34	Male	BSc	International University	8 years
P 14	26	Female	BSc	National Public University	6 months
P 15	30	Female	BSc	National Public University	5 years
P 16	50	Female	BSc	National Public University	12 years
P 17	30	Female	BSc	National Public University	5 years
P 18	37	Female	BSc	National Public University	5 years
P 19	34	Male	High Diploma	National Public University	7 years
P 20	30	Female	MSc	National Public University	4 years
P 21	30	Female	High Diploma	National Public University	6 years
P 22	45	Male	Ph.D	National Public University	18 years
P 23	26	Female	BSc	National Public University	2.5 years
P 24	40	Female	MSc	National Public University	15 years
P 25	31	Female	BSc	National Public University	6 years

**Table 3:** Study themes

Theme	Subtheme
Pharmacist’s perceptions about WLDS	Effectiveness Safety Quality of current products Financial Impact on Pharmacy Business
Prescribing pattern of WLDS by physicians	Most commonly prescribed products Prescribing reasons
Pharmacist’s role in providing pharmaceutical care to patients prescribed WLDS	The action of the pharmacist to customers requesting WLDS Patient education on prescribed WLDS Recommendations to improve pharmaceutical care to patients using WLDS Training and/or education of pharmacists about WLDS

### Pharmacist’s perception about the effectiveness of WLDS

Among the participating pharmacists, 16 perceived WLDS as effective in reducing body weight. Of these, 8 believed that all WLDS are highly effective, while the remaining 8 considered only specific supplements (e.g., AB Slim^®^, Safrolean^®^, Lipo 6^®^, and Slimming Advance^®^) to be effective. Conversely, 6 pharmacists regarded all WLDS as ineffective, and 3 viewed supplements as having limited effectiveness. Notably, most pharmacists with more than 5 years of experience (72%) perceived WLDS as highly effective, whereas approximately two-thirds of those with less than 5 years of experience perceived WLDS as ineffective.

“*According to my work in pharmacy*,* I found that supplements are highly effective in reducing patient weight“(P5)*.

“*They are not effective; I consider them as placebo. 90% of patients did not see any change in their weight by the use of DS“(P7)*.

“*Some of the WLDS are effective while others are ineffective. Even the effective one has small benefits*,* such as green tea*,* since people can lose weight only if they use the supplement along with consumption of a healthy diet and exercising regularly*.“(*P 10)*.

### Pharmacist’s perception about the safety of WLDS

Three of the participating pharmacists perceived that WLDS are safe, while five pharmacists perceived that the safety of WLDS is highly dependent on the manufacturing company. On the other hand, 17 participants considered such products to be unsafe; however, only 12 reported the development of side effects by their customers after the use of WLDS, such as GIT problems (*n* = 7), hypoglycemia (*n* = 1); hypotension (*n* = 1); hypertension (*n* = 1); tachycardia (*n* = 3); agitation (*n* = 1); renal stones (*n* = 2); tremor (*n* = 1), dry mouth (*n* = 1), and depression (*n* = 2).

“*Almost all dietary supplements are safe since they contain herbs“(P 1)*.

“*May be safe; but not all of them. Safety depends on the product manufacturing company“(P 10)*.

“*I consider supplements to be unsafe*,* and I don’t recommend them. These supplements can cause various health issues*,* such as hypoglycemia*,* hypertension*,* and tachycardia. Personally*,* I have witnessed individuals developing comorbidities as a result of using these supplements.“(P 9)*.

### Pharmacist’s perception about the quality of WLDS

Nine of the participating pharmacists perceived that the quality of WLDS was poor, while five participants considered these supplements to have good quality. On the other hand, nine participants considered the WLDS to be of good quality only if it is manufactured by a brand company. The last two participating pharmacists considered the quality of WLDS to be moderate.

“ *I don’t trust the quality of 90% of DS because they are imported from unknown sources. Additionally*,* I don’t trust the quality of products that are being promoted on social media*,* especially when a blogger says that the product can help people lose 10 kilos in a month*” *(P 19)*.

“*The quality is satisfactory*,* and I think that such quality has improved in the last years.“(P 8)*.

*“The quality is variable and mainly depends on the manufacturing company. Some products have good quality while others have poor quality“(P 9)*.

### Pharmacist’s perception about the financial impact of WLDS

Nineteen of the participating pharmacists perceived that selling WLDS has a high financial impact on the pharmacy business, while six participants mentioned that the financial impact is moderate.

“*The pharmacy income can increase by selling DS because their price is higher than drugs“(P 11)*.

“*About the sales of DS in my pharmacy*,* I consider them to have a reasonable (not so high) impact on my pharmacy income “(P 7)*.

### Prescribing pattern of WLDS by physicians

Thirteen of the interviewed pharmacists agreed on the idea that the rate of prescribing WLDS is high nowadays, while twelve pharmacists did not have such belief.

“*For weight loss*,* most physicians prescribe supplements more than drugs*. " *(P 16)*.

“*Rarely physicians prescribe DS; they do not prefer supplements because they want to see a rapid reduction in patient’s weight*.“*(P 2)*.

The participating pharmacists reported that the most common prescribing physicians for DSWL are gynecologists, followed by internists, surgeons, and nutritionists (physicians with a specialty in nutrition who have the authority to prescribe medications). Regarding the prescribed WLDS, most participating pharmacists (*n* = 12) reported that commonly prescribed products are those containing herbs such as green tea, Acai berry fruit extract, ginger, green coffee, and ashwagandha. Nine pharmacists reported that products containing amino acids [L-carnitine (*n* = 8) and L arginine (*n* = 1)] are commonly prescribed. Other products that are reported to be commonly prescribed supplements for weight loss include antioxidants (*n* = 3) [such as alpha lipoic acid (*n* = 1), CoQ10 (*n* = 1), and L-12 (linoleic acid)(*n* = 1)] and spirulina (*n* = 1).

**“***According to my experience*,* Green tea is recommended by internists for obese patients with diabetes*,* and by gynecologists for those with obesity due to polycystic ovarian syndrome*” *(P 10)*.

*“In most weight-loss prescriptions*,* I see products containing L-carnitine or AB-Slim. Mainly surgeons prescribe WLDS before doing gastric sleeve surgery. Gynecologists also prescribe WLDS*,* especially for those with polycystic ovarian syndrome” (P 25)*.

Regarding the reason behind prescribing WLDS, most pharmacists reported more than one reason. Thirteen pharmacists reported that physicians prescribe these supplements for aesthetic reasons (i.e., for patients who seek to improve their body appearance). Seven participants mentioned that physicians add WLDS to their prescriptions for physically inactive patients. On the other hand, 20 pharmacists reported that physicians prescribe these supplements for obese patients suffering from health problems such as infertility (*n* = 12), diabetes (*n* = 3), hypertension (*n* = 2), and joint pain (*n* = 3). Finally, two pharmacists reported that the main reason for prescribing WLDS is the deal of the physician with the supplement manufacturing company. Meanwhile, most participants reported that WLDS were commonly prescribed to female patients (*n* = 21), and only 4 participants reported that such supplements were widely prescribed to males.

*“WLDS are mostly prescribed for females because obesity is more common in females. Besides that*,* females are usually seeking beauty and fitness” (P 16)*.

*“WLDS are commonly prescribed for females with PCOS. They are also prescribed for patients with chronic diseases such as diabetes and hypertension who are willing to lose their weight.“(P 24)*.

“*Physicians usually prescribe supplements for females. They prescribe only items with deals from pharmaceutical companies.* “(*P 2*).

### Action to customers requesting WLDS

Regarding requests for WLDS by customers, seven of the participating pharmacists reported that they usually assess the case before dispensing the requested WLDS. Meanwhile, six participants reported that they usually dispense the requested supplement without assessment of the patient case; however, only three of them reported that they provide their customers with education on the usage of the dispensed supplement. On the other hand, seven pharmacists revealed that they do not agree to dispense these products because of their lack of efficacy and/or safety. The last five pharmacists mentioned referring any patient requesting WLDS to the physician directly.

*“For any customer requesting WLDS*,* I refer him/her to a nutritionist “(P 11)*.

*“Before dispensing the requested product*,* I assess the patient’s case by reviewing the patient’s medical history for any chronic diseases to determine the suitability of the supplement.” (P 16)*.

*“I usually dispense the supplement requested by my pharmacy customers and surely provide the customer with all the instruction about that supplement including its dose and the best time to take “(P 12)*.

### Patient education on the dispensed WLDS

Six of the participating pharmacists (*n* = 6) reported that they usually provide their customers with the necessary information about the prescribed supplements. In this regard, the most commonly mentioned educational information by study participants to their patients include side effects of the WLDS (*n* = 8), method of administration regarding diet and time of the day (*n* = 8), the duration of treatment (*n* = 3), frequency of using the supplements (*n* = 1), and the WLDS dose (*n* = 1). Meanwhile, none of the participating pharmacists provided their customers with all of the above information. On the other hand, 18 pharmacists advised their patients about the need to conduct non-pharmacological measures (such as physical exercise and/or diet) along with the WLDS to ensure weight loss. Worth to note that all participants who perceived WLDS to be ineffective were advising customers about these non-pharmacological measures, while only five of the eight pharmacists (62.5%) who considered WLDS to be effective were providing their customers with education about the necessity of lifestyle changes.

*“When I dispense WLDS*,* I often tell my customer that DS alone is not enough for reducing weight and it does not have a magic effect without dietary restrictions and physical exercise. I also educate the patient about the way of administration and duration of using the DS. “(P 11)*.

*“I educate the patient about the need to take the supplement before meals and the number of times to take the supplement” (P 16)*.

Regarding the barriers for educating customers about the prescribed WLDS, 18 of the interviewed pharmacists reported at least one barrier. These barriers include customers feeling that they have sufficient knowledge about the WLDS (*n* = 2), limited trust in the pharmacist’s competence (*n* = 4), low educational level of the patient (*n* = 6), promotion of the product in social media (*n* = 5), patient’s busy schedule (*n* = 1) and lack of pharmacists’ trust in the efficacy of the requested WLDS (*n* = 1).

“*Patients just want the pharmacist to dispense the prescription because they prefer to get education about their treatment from their physician” (P 5)*.

*“The main barrier to providing patient education is the patient himself*,* especially if the patient is in a hurry when his car is parked in the street “(P 3)*.

### Recommendations to improve pharmaceutical care for patients using WLDS

To improve the role of pharmacists in providing optimum pharmaceutical care to customers using WLDS, the participating pharmacists recommended assessing the quality of such products before marketing them in pharmacies (*n* = 14); conducting research on the WLDS available in the Iraqi market to confirm their efficacy and safety (*n* = 10); conducting pharmacist led-education to physicians about WLDS (*n* = 1); conducting educational programs about WLDS to pharmacists (*n* = 1); encouraging pharmacists to provide patient education about the dispensed WLDS (*n* = 3); obligating supplements manufacturing companies to add details about their product (i.e., ingredients, safety, and instructions for use) (*n* = 3); and conducting pharmacist-led awareness programs to general population about WLDS (*n* = 3).

*“I think it is important to oblige the manufacturing company to put a label on its product containing details about its ingredients*,* side-effects*,* and instruction for use*,* to help pharmacists providing optimum education to consumers. In addition*,* I encourage conducting randomized clinical trials on these supplements to confirm their efficacy for weight loss.” (P 12)*.

*“Supplements must be examined before they become available in pharmacies. Therefore*,* only high-quality products will be dispensed to patients. I also recommend conducting awareness programs for the general population*,* to alarm them that promoting a DS in social media don’t mean that this product has a guaranteed efficacy and safety. " (P 11).*

### Training and/or education of pharmacists about WLDS

Most (*n* = 23) participating pharmacists did not get training or education about WLDS and DS in general. On the other hand, only two pharmacists got information about DS through an educational meeting sponsored by a manufacturing company.

“*The only educational thing about supplements that I had received was the educational meetings with physicians about these supplements that were sponsored by specific companies*.” *(P 5)*.

*“Unfortunately*,* I didn’t receive any training or education about supplements” (P 3)*.

## Discussion

Most participating pharmacists in the present study were young females holding a BSc degree in pharmaceutical sciences. Similar studies conducted among Iraqi pharmacists have reported the same demographic pattern [33, 34]. Furthermore, this sample is highly representative of pharmacists in Iraq, as many pharmacy colleges have been recently established in the past decade and are now admitting large numbers of students. Additionally, the majority of graduated pharmacists are females, and only a small proportion of Iraqi pharmacists hold advanced academic degrees in pharmaceutical sciences [35].

This study showed that approximately two-thirds of the participating pharmacists perceived WLDS as effective in reducing body weight; however, half of those considered all WLDS to be highly effective, while the other half believed that only specific products are effective. Meanwhile, the current literature has not shown any evidence to support such effectiveness [17, 36]. This contradiction with scientific evidence may indicate that the knowledge of current study participants about WLDS is either limited or provoked by the action of medical promotion about these products [37]. On the other hand, most pharmacists with more than 5 years of working experience perceive WLDS to be highly effective, in contrast to those with less than 5 years of experience. This can be explained by the fact that pharmacists with more years of experience typically have a longer period since graduation. During this extended period, there may be a gradual loss of medical and scientific knowledge. Consequently, these pharmacists may be more susceptible to non-scientific promotional influences on such products, which can negatively impact their decision-making.

Regarding the perception of pharmacists about the safety of WLDS, most of them reported that the majority of their customers did not tolerate these supplements and suffered mainly from GIT side effects, depression, and cardiovascular problems (tachycardia and hypertension). There are two main possible reasons for developing these side effects. The first one is related to the usage of ephedra (one of the most common herbs used to lose weight) in WLDS [38], which can cause GIT upset and cardiac side effects [39, 40]. The second reason is the adulteration of these supplements with drugs such as sibutramine [38], which is known to cause cardiac side effects and even depression [41]. The latter reason is highly expected due to Iraq’s weak quality control system, as most participating pharmacists in the current study mentioned.

Regarding the quality of WLDS, most participating pharmacists harbor doubts about products’ quality, especially for products that are not produced by reputable companies. The presence of numerous substandard and counterfeit products in the Iraqi private sector may enhance pharmacists’ doubts about the quality of WLDS in the Iraqi market [42].

Regarding the financial impact of selling WLDS, the results of this study showed that most pharmacists agreed on the high impact of selling such products on their pharmaceutical business. This high financial impact may be attributed to the high cost of DS in Iraq and the high prevalence of obesity among Iraqis, which may result in expanding the sale of such products [4, 43]. This expansion in supplement use is consistent with the global increase in the nutraceuticals and supplements market; such a market worth almost $353 billion USD in 2019 [44].

The results of the current study showed that more than half of the interviewed pharmacists reported that prescribing WLDS is a common practice nowadays. This perception from pharmacists may suggest that physicians are possibly convinced of the efficacy of WLDS, especially L-carnitine-containing products, leading to their frequent prescription.

This high prescribing rate for L-carnitine could be attributed to the availability of some research articles that support the efficacy of L-carnitine in weight loss [45]; these researches may encourage physicians to prescribe these products [45]. However, strong scientific evidence for the efficacy of L-carnitine is lacking [46], and thus, there may be other reasons behind the high prescribing rate of WLDS among Iraqi physicians. In this regard, the results of this study showed that some pharmacists perceived that the pharmaceutical promotion and deals on such products by pharmaceutical companies may be the main trigger for prescribing WLDS. Meanwhile, dealing with pharmaceutical companies may affect the independent decision-making of physicians, thus endangering the quality of patient care, reducing patients’ trust in physicians and the health system, and increasing healthcare costs [47].

Regarding the prescribing of WLDS, this study showed that most of the participating pharmacists mentioned that these supplements are commonly prescribed to female patients. This result is somewhat reasonable since obesity is more common in females, and females are more likely to seek beauty and attractiveness [48, 49]. On the other hand, most pharmacists participating in the current study reported that WLDS are prescribed for patients with health problems such as infertility, diabetes, hypertension, and/or rheumatological problems. These reasons were reported as a main factor for using supplements in other studies that conducted in Canada and Iran [50, 51].

In questioning the interviewed pharmacists about their actions toward customers requesting WLDS, only a minority reported assessing the patient’s case before dispensing the requested WLDS. Poor assessment of the patient case is also critiqued in other studies evaluating Iraqi pharmacists’ role in managing simple illnesses [52, 53]. In addition, most of the participating pharmacists in the current study did not dispense the requested WLDS to their customers. This behavior of pharmacists may indicate their limited conviction in the requested supplement due to limited scientific evidence for product efficacy and poor product quality [54].

The results of the present study showed that only a few of the interviewed pharmacists reported that they usually educate their customers with the necessary information about the prescribed WLDS. Meanwhile, the most commonly mentioned educational points about the dispensed supplement were the side effects, duration of treatment, and method of administration, whereas the dose, dosing frequency, and interactions with the WLDS were the least mentioned. Similarly, other studies conducted in Baghdad, Iraq, found that pharmacists rarely educate their patients about the dispensed product and mainly focus their education process on the method of product administration [19, 55]. On the other hand, many of the participating pharmacists provided their customers with advice about necessity of lifestyle modification; however, it is worth to note that such advice is more commonly given to customer by pharmacists who considered WLDS to be ineffective.

According to the results of this study, all participating pharmacists identified patient-related factors—such as patients’ perceived sufficient knowledge about WLDS, limited trust in pharmacists’ competence, low health literacy, and busy schedules—as primary barriers to effective patient education. Similar patient-related barriers have been reported in studies from developing countries such as Sudan and Ethiopia [56, 57]. Meanwhile, pharmacist-related barriers such as limited pharmacists’ competence, time, and interest in patient education were significant barriers to patient education in the aforementioned studies. Anyhow, pharmacists participating in this study did not report most of these barriers. It is unclear whether these barriers are absent or pharmacists intentionally neglected to report them due to the bias in social desirability that may result from the face-to-face interviews with pharmacists in this qualitative study [58].

However, the latter explanation may be more plausible, as some participating pharmacists indirectly critique their own competence by noting that patients lack trust in pharmacists’ abilities and by emphasizing the need to participate in continuous medical education programs to enhance their skills. This aligns with findings from other countries where limited pharmacist competence has been identified as a barrier to patient education [59, 60]. Additionally, this study revealed that community pharmacists have not received adequate education or training on WLDS, and that the necessary information about WLDS is not being sufficiently communicated to their customers. Therefore, encouraging community pharmacists to participate in educational programs may help enhance the quality of patient education they provide [61].

To improve the pharmaceutical care to patients using WLDS, most participants in the present study recommended assessing the quality of WLDS before marketing these products in pharmacies and conducting research on the products available in the Iraqi market to confirm their efficacy and/or safety. These recommendations are highly expected since most pharmacists participating in the current study considered the quality of WLDS available in the Iraqi pharmaceutical market to be poor. Other pharmacists recommended conducting pharmacist-led educational programs to increase the general public’s awareness of DS. This recommendation seems necessary to combat the negative effect of misleading advertisements of WLDS in social media and satellite channels [62, 63].

The findings of this study should be interpreted within certain limitations. First, the sample size was relatively small and was drawn exclusively from a single governorate, which may restrict the ability to generalize these results to other regions or populations. A larger, more diverse sample across multiple areas would enhance the external validity of the findings. Second, participants’ claims, such as their perceptions or reported behaviors, may be affected by social desirability and recall biases. These issues are inherent to the qualitative design of the study and are commonly observed in qualitative research conducted in Iraq [21, 34, 64]. Third, most of the enrolled pharmacists were relatively young and had limited professional experience; this demographic factor could influence their perspectives and may not fully represent the views of more experienced practitioners, potentially affecting the breadth of insights captured. Fourth, the process of translating interviews from Arabic to English introduces the potential for language nuances or cultural contexts to be lost or distorted, which could impact the accuracy of data interpretation. Although such linguistic challenges are common in studies conducted in Arab countries, they may nonetheless influence the fidelity of participants’ expressions. Finally, the inclusion of pharmacists who currently utilize WLDS in their practice may introduce response bias, as their perspectives on the efficacy or safety of these products could be influenced by personal financial incentives or vested interests, thus possibly skewing the results. Acknowledging these limitations is crucial for contextualizing the findings and guiding future research. Despite these limitations, this study marks the first exploration of WLDS from the pharmacists’ point of view in Iraq. The unique findings may pave the way for further research assessing the quality, safety, and effectiveness of dietary supplements in the Iraqi market.

In conclusion, participating pharmacists reported that WLDS products are most commonly prescribed for patients with infertility and chronic diseases. Many pharmacists have expressed concerns regarding the safety, efficacy, and quality of the WLDS products available in the market. These observations highlight the importance of further research to comprehensively evaluate the safety, efficacy, and quality of WLDS before widespread market introduction. Additionally, existing gaps in pharmacists’ training and patient education highlight the need for ongoing medical education programs and specialized training. These measures may help improve pharmacists’ communication skills, support evidence-based counseling, and increase awareness of WLDS risks, which could contribute to enhancing pharmaceutical care for WLDS users and potentially benefit patient outcomes.

## Data Availability

No datasets were generated or analysed during the current study.

## Appendix

### Exploring the knowledge, perceptions, and dispensing practices of weight loss dietary supplements by community pharmacists in Baghdad, Iraq

#### Part 1: demographic data of participants

Age:

Gender:

College that you graduated from:

Academic degree:

Location of pharmacy:

Working experience:

#### Part 2: semi structured interview guideline

1. What is your perception about the efficacy of weight loss dietary supplements (WLDS)? Can you explain by giving some examples?
2. What is your perception about the safety of WLDS? Can you explain?
3. What do you think about the current prescribing rate of WLDS by physicians? To which patients these supplements are mainly prescribed?
4. What is the main driver for prescribing of such products by physicians?
5. What are the most commonly WLDS that recorded in physicians prescriptions? Why?
6. As a pharmacist, do you think you have to provide customers with certain information about the WLDS that you dispense? Probes: can you give me an example for the education that you provide to your customers?
7. What are the barriers to educate patients about WLDS?
8. What is your action regarding a customer who asks you about a specific WLDS? 
9. What is the impact of selling WLDS on pharmacy business?
10. What is your opinion about the quality of the available WLDS in the Iraqi market?
11. What training or education have you had about WLDS and DS in general? If yes, give me some more details about it.
12. What is your recommendation to improve the pharmaceutical care to customers using these supplements?
13. Any further comments?
